# A comprehensive framework for functional diversity patterns of marine chromophytic phytoplankton using *rbc*L phylogeny

**DOI:** 10.1038/srep20783

**Published:** 2016-02-10

**Authors:** Brajogopal Samanta, Punyasloke Bhadury

**Affiliations:** 1 Integrative Taxonomy and Microbial Ecology Research Group, Department of Biological Sciences, Indian Institute of Science Education and Research Kolkata, Mohanpur-741246, Nadia, West Bengal, India

## Abstract

Marine chromophytes are taxonomically diverse group of algae and contribute approximately half of the total oceanic primary production. To understand the global patterns of functional diversity of chromophytic phytoplankton, robust bioinformatics and statistical analyses including deep phylogeny based on 2476 form ID *rbc*L gene sequences representing seven ecologically significant oceanographic ecoregions were undertaken. In addition, 12 form ID *rbc*L clone libraries were generated and analyzed (148 sequences) from Sundarbans Biosphere Reserve representing the world’s largest mangrove ecosystem as part of this study. Global phylogenetic analyses recovered 11 major clades of chromophytic phytoplankton in varying proportions with several novel *rbc*L sequences in each of the seven targeted ecoregions. Majority of OTUs was found to be exclusive to each ecoregion, whereas some were shared by two or more ecoregions based on beta-diversity analysis. Present phylogenetic and bioinformatics analyses provide a strong statistical support for the hypothesis that different oceanographic regimes harbor distinct and coherent groups of chromophytic phytoplankton. It has been also shown as part of this study that varying natural selection pressure on form ID *rbc*L gene under different environmental conditions could lead to functional differences and overall fitness of chromophytic phytoplankton populations.

Ocean productivity largely refers to the biological primary production in euphotic zone^1^. Photosynthetic carbon fixation by marine phytoplankton contributes about half of the global primary production in contemporary ocean^2^. Phytoplankton with high species diversity (>20,000 species) and wide range of size variations represents the most successful primary producer across global oceanographic regimes. In contrast to its counterpart in terrestrial environment, species diversity of phytoplankton is 12-fold lower but taxonomic division is 8 orders of magnitude higher than terrestrial plants^3^. Among phytoplankton, chromophytes contribute approximately 50% of the total oceanic primary production^4^. These unicellular microalgal groups comprise of 15 taxonomic classes which are represented by four major divisions^5^ i.e. Heterokontophyta, Cryptophyta, Haptophyta and Rhodophyta. Ecologically, taxonomic diversification emphasizes the importance of marine environment in controlling structure and function of chromophytic phytoplankton communities.

Satellite ocean color data provide a general view of total phytoplankton distribution across different marine environment^2^,^6^. Analysis of phytoplankton functional types from optical data and *in situ* measurement show that picoplanktonic prokaryotic photoautotrophs are the most successful primary producers in oligotrophic open ocean environment, whereas chromophytes dominate bulk of phytoplankton assemblage in marginal ecosystems such as estuarine and coastal environments^4^. Annual phytoplankton primary production (APPP) in the world’s estuarine-coastal ecosystems show variation between ecosystems, followed by spatial level variability within ecosystems and temporal scale variability between years of a particular ecosystem^7^. In the last decade, world’s ocean surface chlorophyll data derived from satellites show that micro-phytoplankton (mostly diatoms) contribute about 70% of the total primary production in coastal upwelling systems^8^. Furthermore, abundant supply of regenerated nutrients enhance new production in tropical and subtropical coastal upwelling environments^8^,^9^. Therefore, observed high primary productivity in coastal ecosystems motivated us to explore the hidden diversity of key primary producer from different ecologically significant ecosystems including upwelling, seasonal bloom site, river plume, and coastal mangrove environments.

The rate limiting enzyme, ribulose-1, 5-bisphosphate carboxylase/oxygenase (RubisCO), found across three domains of life *i.e.* archaea, bacteria, and eukarya, is principally involved in sequestration of carbon dioxide from environment by reductively assimilating into organic carbon within cellular biomass^10^,^11^,^12^. Based on amino acid sequences homology and phylogeny^13^,^14^, four known forms of RubisCO (forms I, II, III and IV) are found in nature. Moreover, form I RubisCO can be further sub-divided into two major subgroups^14^: green (cyanobacteria, green algae and plants) and red (phototropic bacteria and chromophytic phytoplankton) lineages. Green lineage can be further subdivided into forms IA and IB and red lineage into forms IC and ID^11^. Most of the non-green phytoplankton, also termed as chromophytic phytoplankton, contains form ID RubisCO^14^.

Traditionally, bright field and electron microscopy are widely used for taxonomy and biodiversity assessment of chromophytes in natural assemblages^15^,^16^. Recently, details of species diversity and taxonomic inventories using molecular markers, fine scale morphological characteristics, and cross experiments revealed improved resolution of chromophytic phytoplankton species diversity^17^,^18^. In the last two decades, form ID *rbc*L has been extensively used as a reliable phylogenetic marker for assessment of functional biodiversity of chromophytic phytoplankton from different coastal ecoregion based mainly on clone library and sequencing approach^19^,^20^,^21^,^22^,^23^,^24^. However, to date global distribution patterns of *rbc*L phylotypes as proxy of chromophytic phytoplankton assemblages across different oceanographic ecoregions remain largely unknown. Moreover, phylogenetic analyses of *rbc*L gene suggest that sequences from one environment tend to cluster with another environment^24^, but the significance of such clustering is yet to be comprehensively investigated. We hypothesize that different oceanographic regimes harbor distinct and coherent groups of chromophytic phytoplankton or specific clade of chromophytes display biogeographic patterns.

To understand the global patterns of functional diversity of chromophytic phytoplankton, robust phylogenetic analysis based on functional gene marker (*rbc*L) were undertaken with uncultured form ID *rbc*L sequences retrieved from GenBank database across seven different ecologically significant oceanographic regions representing tropical and subtropical gyres. Therefore, the main objective of this study were: (1) to understand distribution patterns of uncultured chromophytic phytoplankton assemblages across different oceanographic ecoregions globally based on form ID *rbc*L deep phylogeny and bioinformatics analyses, (2) to detect novel form ID *rbc*L sequence types and their distribution patterns across different oceanographic ecoregions, and (3) to gain an insight on the role of selection pressure on *rbc*L gene for functional attribution of RubisCO enzyme of chromophytic phytoplankton in studied environments. Together, these robust phylogenetic and bioinformatics analyses based on global form ID *rbc*L datasets will provide a benchmark in terms of changes in functional diversity of chromophytic phytoplankton assemblages according to the type of ecosystem and associated environmental conditions.

## Results

### Overview of the form ID *rbc*L sequence diversity

We compiled and aligned 2624 uncultured form ID *rbc*L sequences representing seven different oceanographic ecoregions globally. Majority of the *rbc*L sequences were generated from East China Sea (27%), Sundarbans mangrove ecosystem (25%), and South China Sea (20%) with respect to total number of sequences considered in this study. In the final alignment within overall dataset, 1112 sequences (42%) were found to be unique (Table 1). Average pairwise comparison among unique sequences from each ecoregion showed that sequences were most similar within each of the East China Sea (94%) and Sundarbans (96%) dataset (Supplementary Fig. S1). Average G+C percentage of this partial segment of *rbc*L gene also varied between studied sites (Supplementary Fig. S2). For example, Monterey Bay *rbc*L dataset showed highest average G+C percentage (41.25%), whereas it was lowest in case of ALOHA stations (38.52%). To understand the distribution patterns of uncultured chromophytic phytoplankton across seven ecoregions, form ID *rbc*L sequences were grouped into OTUs up to 85% amino acid identity level (Table 1). Sequences from Sundarbans mangrove ecosystem (SB) has the highest number of observed unique OTUs as well as 99% amino acid level identity; whereas East China Sea (EC) has highest number of OTUs at 98%, 97%, 95% and 85% amino acid identity levels (Table 1). However, some of this apparent diversity could be also due to contribution from PCR error. One PCR error in 10^4^ bases would result in 99.99% similarity after 1 cycle^20^. Therefore, the percentage of similarity between a true and artifact sequence due to PCR error would be 99.7% after 35 PCR cycles. Hence, the clones that are >99.7% similar are considered as identical sequences. Even considering for ~5 PCR or sequencing error in each 554 bp *rbc*L fragment (corresponding to the 99% identity level), there was only 10% decrease in OTUs from unique to 99% amino acid identity level. Moreover, rarefaction analyses at different identity levels of amino acid indicated that observed OTU numbers are yet far from saturation at 99% identity level in all the targeted ecoregions (Supplementary Fig. S3). It is important to note that no microscopic data were available from these samples to compare with *rbc*L clone library datasets for each of the targeted ecoregion. However, the degree of genetic diversification without morphological consideration for species demarcation reflects gross functional diversity, but these OTU numbers estimated the overall diversity of form ID *rbc*L sequences as proxy of chromophytic phytoplankton across studied ecoregions.

### Global phylogeny of uncultured form ID *rbc*L sequences

**Figure 1:**
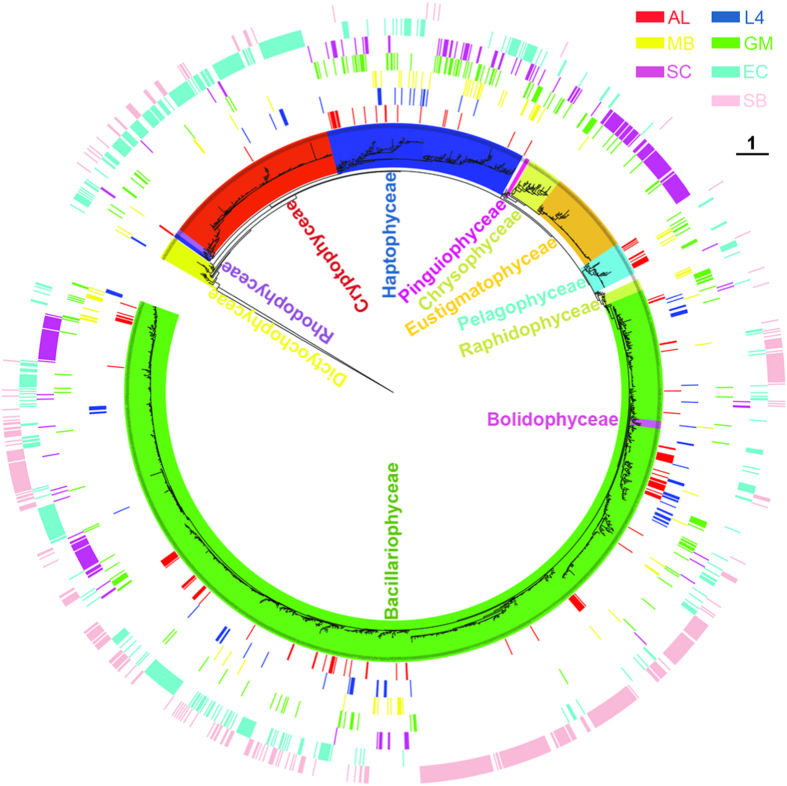
RAxML phylogeny of uncultured form ID *rbc*L sequences and their distribution patterns. OTU grouping was not undertaken before phylogenetic tree construction. Colored bars in the outer rings correspond to the ecoregion assignment for each sequence. Clade and branch color codes indicate the taxonomic class assignment of the *rbc*L sequences. AL = ALOHA, L4 = L4 site of Western English Channel, MB = Monterey Bay, GM = Gulf of Mexico, SC = South China Sea, EC = East China Sea, and SB = Sundarbans mangrove ecosystem.

Phylogenetic analysis with 2624 uncultured form ID *rbc*L sequences from seven distinct ecoregions recovered 11 major clades of chromophytic phytoplankton in varying proportions (Fig. 1). These eleven major clades represented 11 different taxonomic classes of chromophytic phytoplankton. Details of the cultured chromophytic phytoplankton *rbc*L sequences used in the present phylogenetic analysis to annotate taxonomic affiliation of uncultured form ID *rbc*L sequences were provided in Supplementary Table S1. Taxonomic class specific diversity of uncultured form ID *rbc*L sequences was highest in Gulf of Mexico (9 classes), whereas lowest in ALOHA station of North Pacific Subtropical Gyre (only 4 classes) (Supplementary Table S2). Global phylogenetic analysis showed that Bacillariophyceae (Diatoms), Cryptophyceae, and Haptophyceae like *rbc*L sequences were the major chromophytic phytoplankton groups detected in each of the seven targeted ecoregions. Diatom like *rbc*L sequences were by far the most detected chromophytic phytoplankton signature from all the ecoregions, followed by Haptophyceae and Cryptophyceae, but their community structure varied across studied ecosystems (Fig. 1). For example, genera such as *Thalassiosira, Chaetoceros,* and *Phaeocystis* like *rbc*L sequences were ubiquitous based on global phylogeny, whereas minor taxonomic classes of chromophytic phytoplankton such as unicellular Rhodophyceae (order Porphyridiales), Bolidophyceae and Pinguiophyceae like *rbc*L sequences were restricted to certain oceanographic regimes (Fig. 1, Supplementary Table S2).

The ANOSIM (R = 0.359, P < 0.001) and AMOVA (Fs >1, P < 0.001) analyses for overall rbcL sequence datasets showed significant difference in chromophytic phytoplankton community structure from one ecoregion to another. Pairwise ANOSIM (as in all cases R >0.1 and P < 0.001) and AMOVA (as in all cases Fs >1, P <0.001) analyses also confirmed that chromophytic phytoplankton community structure varied significantly from one ecoregion to another except for GM-L4 and GM-MB (Supplementary Table S3). In addition, each pair of seven different ecoregions were significantly different from each other based on LIBSHUFF test (P <0.001) except for MB-EC (P = 0.311) and SC-GM (P = 0.668) in terms of ID *rbc*L sequence data types.

### Global phylogeny of novel form ID *rbc*L sequences

**Figure 2:**
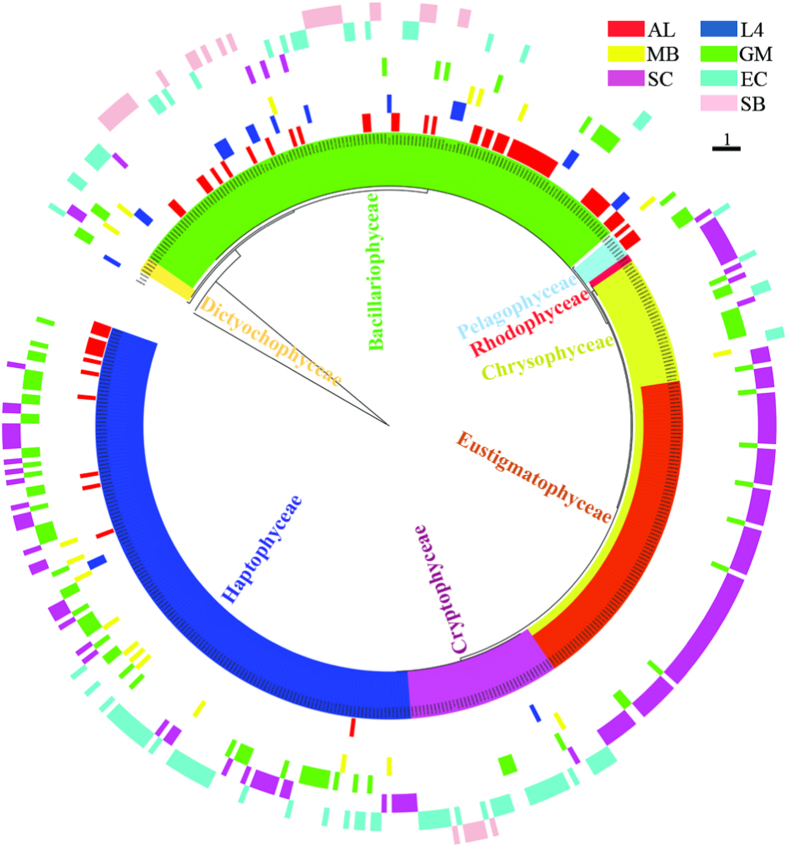
Phylogeny of novel uncultured form ID *rbc*L sequences and their distribution patterns. OTU grouping was not undertaken before phylogenetic tree construction. Colored bars in the outer rings correspond to the ecoregion assignment for each sequence. Based on blastp analysis, sequences that showed ≤95% identity with cultured chromophytic phytoplankton *rbc*L sequences available in the published databases, considered as novel *rbc*L sequences. Clade and branch color codes indicate the taxonomic class assignment of chromophytic phytoplankton. AL = ALOHA, L4 = L4 site of Western English Channel, MB = Monterey Bay, GM = Gulf of Mexico, SC = South China Sea, EC = East China Sea, and SB = Sundarbans mangrove ecosystem.

In blastp result, sequences that showed ≤95% identity with cultured chromophytic phytoplankton *rbc*L sequences available in published databases, were considered as novel uncultured form ID *rbc*L sequences. A total of 455 novel unique sequences were recovered from the analyzed datasets. Phylogenetic analysis with those novel sequences recovered eight classes of chromophytic phytoplankton representing all the ecoregions in varying proportion (Fig. 2). The South China Sea dataset was represented by highest number of novel unique sequences (about 90%). It is also important to note that about 64% of total novel unique sequences of South China Sea belonged to Eustigmatophyceae and Chrysophyceae. Novel *rbc*L sequences representing Haptophyceae and Bacillariophyceae like novel sequences were most frequently detected chromophytic phytoplankton signature, followed by Eustigmatophyceae, Chrysophyceae, and Cryptophyceae. Moreover, Eustigmatophyceae like novel rbcL sequences were only detected from Gulf of Mexico and Daya Bay of South China Sea ecoregions (Fig. 2).

The ANOSIM (R = 0.295, P < 0.001) and AMOVA (Fs = 22.77, P < 0.001) analyses with total novel *rbc*L sequences from targeted ecoregions showed that each ecoregion harbor significantly distinct chromophytic phytoplankton which are yet to be explored at the morphological and physiological level based on cultured approaches.

### Different ecoregions harbor distinct and coherent form ID *rbc*L sequence types

**Figure 3:**
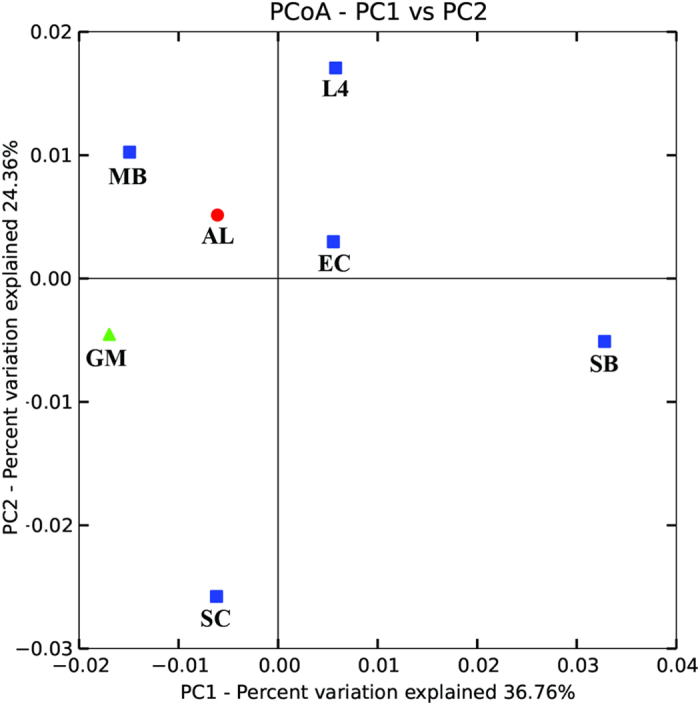
Principal Coordinate Analysis (PCoA) of weighted normalized UniFrac distances of *rbc*L sequences across seven different oceanographic ecoregions of the world. First two components explained about 60% of total variance in the *rbc*L dataset. Unifrac analysis was conducted based on the best scoring RAxML tree. AL = ALOHA, L4 = L4 site of Western English Channel, MB = Monterey Bay, GM = Gulf of Mexico, SC = South China Sea, EC = East China Sea, and SB = Sundarbans mangrove ecosystem.

**Figure 4:**
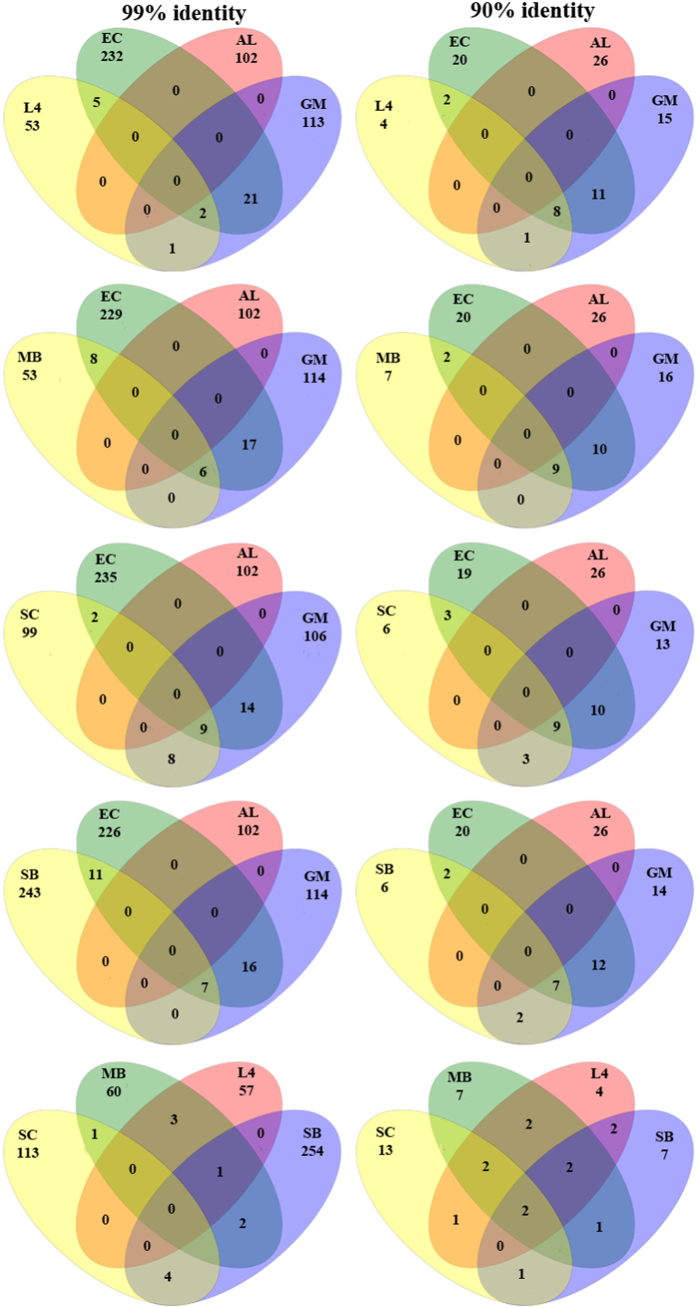
Venn diagrams of form ID *rbc*L OTUs distribution patterns at 99% and 90% amino acid identity level across seven targeted ecoregions. AL = ALOHA, L4 = L4 site of Western English Channel, MB = Monterey Bay, GM = Gulf of Mexico, SC = South China Sea, EC = East China Sea, and SB = Sundarbans mangrove ecosystem.

We used UniFrac distances from each set of sample to better understand the genetic heterogeneity of form ID *rbc*L sequence types among seven different oceanographic ecoregions. Principal Coordinate Analysis (PCoA) based on UniFrac distance matrix revealed that chromophytic phytoplankton community structure of each ecosystem was different from others across the first two components which explained about 60% of total variance (Fig. 3). Andy Martin’s phylogenetic (P) tests of each pair of ecoregions’ *rbc*L sequence types were significantly different from one another (P < 0.001). UniFrac significance test based on unique branch length present in each ecoregion showed that ALOHA (AL), English Channel (L4), and Gulf of Mexico (GM) harbored significant numbers of unique branch length in the phylogenetic tree (P < 0.01). Pairwise UniFrac significance test among seven targeted locations showed that unique branch length of *rbc*L sequences of Sundarbans mangrove ecosystem (SB) was significantly different from the rest (Supplementary Fig. S4). Moreover, majority of OTUs of each targeted ecoregion was found to be restricted within respective ecosystem, whereas some OTUs were shared by two or more habitat types at 99% and 90% identity level of amino acid (Fig. 4). It is important to note that OTUs of open oceanic time series station ALOHA were unique at any cutoff level with respect to other targeted ecoregions (Fig. 4).

Phylip-formatted distance matrix based β-diversity analysis among seven targeted ecoregions indicated that *rbc*L sequence types were strongly partitioned between open ocean and coastal ecosystems (Supplementary Table S4). Pairwise comparisons of the number of OTUs shared between any two habitats (using Jaccard similarity coefficient) showed that ALOHA station had no common OTUs with other targeted coastal ecoregions, but there were shared OTUs between any two of the six coastal ecoregions except between L4 and SC at 99% identity level of amino acid (Supplementary Table S4). Statistically, the proportions of shared OTUs significantly varied from one coastal ecoregion to other at different identity level of amino acid (Fig. 4, Supplementary Table S4).

### Functional diversity of form ID *rbc*L sequences in different ecoregions

**Figure 5:**
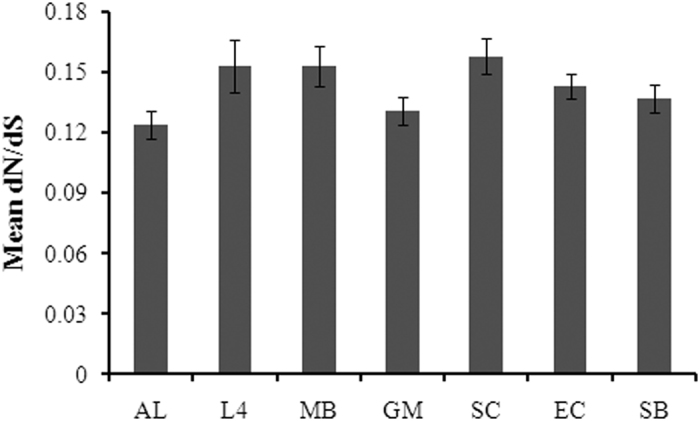
Selective pressure (dN/dS) on form ID *rbc*L sequences from seven different ecoregions as calculated by the SLAC algorithm. Error bars indicate upper and lower 95% confidence intervals. AL = ALOHA, L4 = L4 site of Western English Channel, MB = Monterey Bay, GM = Gulf of Mexico, SC = South China Sea, EC = East China Sea, and SB = Sundarbans mangrove ecosystem.

The existence of distinct and coherent pattern of form ID *rbc*L sequences in each of the targeted ecoregion could be explained due to varying selection pressures on the function of RubisCO enzyme. To gain an insight into the strength of selective pressures acting on *rbc*L, ratio of non-synonymous to synonymous substitutions (dN/dS) were calculated from each dataset separately. It is important to note that out of total 2624 form ID *rbc*L sequences, only 12 position (about 6.5%) were completely conserved across the entire *rbc*L dataset alignment (184 amino acid length). The dN/dS ratio varied from 0.124–0.158 using unique form ID *rbc*L nucleotide sequences individually from each dataset (Fig. 5). Moreover, there was less evidence of positive selection at any individual codon position in each dataset. For example, dataset of L4, SC, EC and SB showed 1, 2, 2, and 3 positively selected codon positions respectively (SLAC algorithm, P < 0.1).

## Discussion

Boyd and Doney postulated the rule of universal distribution and local selection of planktonic functional groups across different oceanographic regimes^25^. With respect to higher taxonomic ranks (such as division or class), coherent distribution pattern of *rbc*L phylotypes was revealed from present phylogenetic and bioinformatics analyses. But form ID *rbc*L phylotypes heterogeneity was potentially vast at lower taxonomic ranks (such as genus, species and infra-species) in each of the seven targeted ecoregions which indicated the uniqueness of chromophytic phytoplankton community structure. In the Gulf of Mexico and East China Sea pooled datasets, much higher number of OTUs had been detected at higher cut off level (*i.e.,* above 95% identity at the amino acid level) compared to other ecoregions (Table 1). It is important to note that datasets representing these ecoregions consist of several spatio-temporal diverse sampling points. For example, form ID *rbc*L dataset of Gulf of Mexico were compiled from the East and Southeast Gulf^19^,^21^, Northern Gulf^26^, Florida shelf^19^,^22^, and chlorophyll-rich costal plume area^20^. This could be one of reasons for the detection of higher number of OTUs in Gulf of Mexico and also for East China Sea *rbc*L datasets. On the other hand, *rbc*L sequences of South China Sea were all collected from one bay *i.e.* Daya Bay, and the number of estimated OTUs was much lower compared to Gulf of Mexico and East China Sea. The main aim of this work was not to account for potential spatio-temporal variations of chromophytic phytoplankton community structure in each targeted ecoregion, but to elucidate clade specific chromophytic phytoplankton biogeographic patterns using *rbc*L phylogeny. Moreover, distribution patterns of observed OTUs at different amino acid identity level provided a general estimation of overall uncultured chromophytic phytoplankton community structure across different oceanographic ecoregions. The major findings of this study support our hypothesis that each ecosystem harbor distinct and coherent group of chromophytic phytoplankton. Overall, the present study also suggested that numbers of undiscovered uncultured chromophytic phytoplankton are still potentially vast in these ecoregions.

Diatoms, the most ecologically significant groups of chromophytic phytoplankton, successfully dominate bulk of the phytoplankton assemblages across different ecoregions. Previously, detailed taxonomic inventories using fine-grained morphological characteristics, molecular markers and reproductive isolation studies have revealed global scale to narrow endemic geographical distribution pattern of diatoms^27^,^28^. Our global *rbc*L phylogeny also showed the coherent and distinct distribution patterns of phylotypes within the diatom clade. The coherent distribution pattern of some diatom subclades, for example *Thalassiosira* and *Chaetoceros* like *rbc*L sequences, across different ecoregions may be due to their wide range of physiological or genome plasticity under different environmental conditions thereby resulting in high species diversity. Moreover, discovery of cryptic diversity^29^,^30^ within cosmopolitan diatom genera could be extended to functional level distribution patterns between allopatric populations and ultimate understanding of their ecology. In the present study, ecoregion specific *rbc*L gene heterogeneity within these sub-clades may be due to local selection pressure that ultimately may lead to functional evolution within chromophytic phytoplankton population. On the other hand, several subclades were recovered from certain ecoregions as evident from Figs 1 and 2. For example, *Amphora* and *Halmophora* like *rbc*L sequences were mostly detected from Sundarbans mangrove ecosystem which is characterized by intense vertical mixing of the water column due to strong influence of diurnal tide. As a result of such dynamic nature of this ecosystem, some benthic or tychoplanktonic diatoms contribute a significant role in primary productivity in the water column compared to typical planktonic diatom communities. Another example of ecoregion specific distinct community structure of diatoms is for ALOHA site, an oligotrophic open ocean environment, where about 50% of diatom like *rbc*L sequences showed <95% identity at the amino acid level with cultured diatom sequences available in published databases. Although diatoms are the most thoroughly studied taxonomic class of chromophytic phytoplankton, but evidence of several deeply branched sequences within the diatom clade indicates that numerous species are yet to be discovered across marine environments globally. The present study showed that assemblage patterns of diatoms are strongly correlated with environmental conditions and they overwhelmingly dominate assemblages across studied ecoregions.

Although the influence of environmental variables on chromophytic phytoplankton community structure was not the scope of this study, but these have been extensively discussed from the targeted ecoregions^21^,^31^,^32^,^33^,^34^,^35^. It is evident from the present study that overall chromophytic phytoplankton communities (as form ID *rbc*L phylotypes) in each ecosystem was strongly influenced by local variability of environmental parameters. As a result of such local selection pressure on chromophytic phytoplankton communities, functional genes may evolve leading to wider adaptability of phytoplankton community and ultimately may lead to increased primary productivity in each ecosystem. For example, class specific diversity of chromophytes was less in open ocean oligotrophic ecoregion of ALOHA stations, but species specific diversification within these classes was several magnitudes higher. Chrysophyceae and Eustigmatophyceae like *rbc*L sequences were not detected from open ocean time-series station ALOHA and L4 site of Western English Channel, but these were mainly detected from those ecoregions where influence of fresh water run-off is more, for example, coastal high chlorophyll plume in Gulf of Mexico is formed due to the Mississippi river discharge. It should also be noted that several Chrysophyceae and Eustigmatophyceae like novel *rbc*L sequences are thus far mostly detected in the Daya Bay of South China Sea. Low water exchange rate with coastal water, relatively shallow depth of the water column, and strong influence of Zhujing River in the Daya Bay may favor genus and species level diversification of these two classes.

Haptophyceae like *rbc*L sequences represented second dominant clade in global phylogenetic tree but it constituted the largest clade and represented by novel sequences. Light microscopy is often insufficient to identify Haptophytes^36^ beyond generic level as species identification mostly relies on scale morphology. It is usually inadequate, except for some species (e.g., *Phaeocystis pouchetii*), to identify them up to species level in preserved material^36^. Such kind of taxonomic intractability is also associated with the other classes of chromophytic phytoplankton, for example in case of Cryptophyceae^37^ and Raphidophyceae^37^. It is also important to note that minor taxonomic classes of chromophytic phytoplankton such as unicellular Rhodophyceae (order Porphyridiales), Bolidophyceae, and Pinguiophyceae like *rbc*L sequences were only detected in certain oceanographic regimes. But it is possible that these classes are not exclusive to these ecosystems. As evident from rarefaction analysis, number of OTUs from each ecoregion were far from saturation at the 99% amino acid level identity. For example, Synurophyceae and Phaeothamniophyceae like *rbc*L sequences were not detected in the global phylogenetic tree. Therefore, culture establishment and detail taxonomy study of these minor classes of chromophytic phytoplankton are still overlooked in the field of phycology. Further sequencing effort including application of next generation sequencing may increase the chances of detection of their signature from a wide range of oceanographic realms. Considering all these points together, present phylogenetic analyses suggest that integrated taxonomic approach (using light microscopy, electron microscopy and multigene phylogeny) must be used to explore unknown diversity of chromophytic phytoplankton functional groups. The present study also indicated that further sequencing effort must be undertaken with culture chromophytic phytoplankton to make the existing *rbc*L sequence databases more robust. While there is a large amount of variation in functional diversity of uncultured form ID *rbc*L sequences, successful culturing of novel uncultured chromophytic phytoplankton from different environments and subsequently their polyphasic taxonomy will help us to increase our understanding about their role in primary production across various coastal and open ocean ecosystems.

In the global *rbc*L phylogeny, Cryptophyceae like sequences were mostly detected from East China Sea and Sundarbans mangrove ecosystem. From these two ecoregions, several Cryptophyceae like sequences showed 100% identity with *Dinophysis fortii* (Dinophyceae or Dinoflagellates) at the amino acid level. In the evolutionary perspective^3^,^38^,^39^, *Dinophysis fortii* temporary acquired the Cryptophycean plastid to continue their autotrophic mode of nutrition. Presently it is difficult to assign as to whether these sequences actually belong to Dinophyceae or Cryptophyceae. But it can be concluded from the present phylogenetic analysis that some heterotrophic Dinophyceae may play an important role in overall primary production in these two ecoregions by transforming their mode of nutrition when favorable environmental conditions support autotrophic growth. Previous studies^31^,^33^ based on microscopic and pigment data analyses showed that Dinoflagellates are one of the major functional group in natural phytoplankton assemblages from these two ecoregions.

As *rbc*L is the catalytic subunit of RubisCO, investigation of natural selection pressure on *rbc*L gene could explain the functional diversity of chromophytic phytoplankton in each of these environments. Moreover, *rbc*L is an ancient gene and has relatively less sequence variability compared to other functional genes such as those involved in ammonium and nitrate metabolism^40^. However, ecosystem-specific selection pressure^41^ always plays a vital role on the functional genes of organismal communities in any natural environment. Here, we wanted to know if natural selection pressure on form ID *rbc*L gene might differ for functional attribution of chromophytic phytoplankton population structure across varied natural environments. As dN/dS ratio is <1 in all cases, the deleterious non-synonymous substitutions in *rbc*L gene were removed from chromophytic phytoplankton population in each of the seven targeted ecoregions through purifying (negative) selection. Our results also indicated that some amino acid substitutions may be raised by positive selection, but not enough to overcome the effects of purifying selection in these environments. For example, highest positive selection pressure was detected in Sundarbans mangrove ecosystem which indicated the improving fitness of the functional enzymes such as RubisCO in chromophytic phytoplankton in this dynamic environment. Overall, different selection pressure on form ID *rbc*L gene in different environmental conditions could lead to functional differences and overall fitness of chromophytic phytoplankton populations in these environments.

In this study, we highlighted the vast magnitude of functional diversity of chromophytic phytoplankton across different oceanographic ecoregions and demonstrated that distinct ecotypes of phylogenetically related form ID *rbc*L sequences were restricted to certain ecosystems. However, with increased sampling of form ID *rbc*L diversity, unknown chromophytic phytoplankton species have begun to emerge. The remarkable advancement in next generation sequencing technology will enable future studies to undertake more meticulous survey of chromophytic phytoplankton diversity from different ecologically significant marine environments. Moreover, our global *rbc*L phylogenetic analyses will be a benchmark dataset for the rapidly expanding field of single cell genomics, metagenomics, and transcriptomics to revolutionize the understanding of biodiversity and ecology of unknown chromophytic phytoplankton.

## Methods

### *rbc*L sequence datasets

Form ID *rbc*L sequences were extracted from databases (GenBank/DDBJ/EMBL/PDBJ) by searching for records identified as environmental samples containing search items “*rbc*L” and “uncultured marine microorganism”, “uncultured eukaryote”, “uncultured phytoplankton”, “uncultured phototropic eukaryote”, or “uncultured marine phototropic eukaryote”. Datasets were downloaded directly from GenBank. We retrieved form ID *rbc*L sequences from seven different ecologically significant ecoregions of the world: ALOHA station (AL), English Channel (L4), Monterey Bay (MB), Gulf of Mexico (GM), South China Sea (SC), East China Sea (EC), and Sundarbans mangrove ecosystem (SB) at the apex of Bay of Bengal (Supplementary Fig. S5). One dataset was generated by Li *et al*.^34^ from station ALOHA, part of the Hawaii Ocean Time-series (HOT) in North Pacific Subtropical Gyre, represents an oligotrophic environment. Dataset of Monterey Bay (MB), a coastal upwelling site on the California coast, and L4 site of Western English Channel (L4), a North Atlantic spring bloom coastal environment, were generated by Bhadury and Ward^22^. Datasets of Gulf of Mexico^19^,^20^,^21^,^23^,^26^ were generated from different regions including Eastern and Southeastern part, West Florida Shelf (UID: 28932298), and high chlorophyll coastal plume regions resulting from Mississippi river discharge. Datasets of South China Sea were from the Daya Bay (UID: 612163157, 612162813), characterized by low rate of water exchange with sea water of South China Sea and the East Guangdong upwelling transports the cold water, leading to thermocline during summer^42^. Datasets of East China Sea^43^,^44^ were generated from Jiaozhou Bay (UID: 38710252, 34538971, 33468242) representing two sites with coordinates; 30.85˚N 122.67˚E (UID: 564813280) and 30.25˚N 123.42˚E (UID: 602620318). Moreover, one dataset was generated from the world’s largest mangrove ecosystem, Sundarbans^24^ (SB), at the apex of Bay of Bengal. It is important to note that all the datasets considered for the present analyses were generated by PCR based clone library approach using same set of primers (i.e., forward primer, 5′-GATGATGARAAYATTAACTC-3′; reverse primer 5′-ATTTGDCCACAGTGDATACCA-3′) except for 46 sequences out of 712 sequences^43^,^44^ representing the East China Sea.

### Form ID *rbc*L clone library preparation from Sundarbans Biosphere Reserve

Previously^24^, ten clone libraries were generated from a macrotidal creek and adjoining estuary of Indian part of Sundarbans which is characterized by a planted patchy mangrove area and strongly influenced by coastal water from the Bay of Bengal. To elucidate the overall chromophytic phytoplankton assemblages in other part of Indian Sundarbans mangrove ecosystem, twelve additional *rbc*L clone libraries were generated across different geographic locations of Indian part of Sundarbans Biosphere Reserve (SBR) which is a protected pristine natural mangrove area as part of the present study (Supplementary Table S5). Environmental DNA was extracted from surface water sample of each station using standard published protocol^45^. Partial *rbc*L gene fragment (554 bp) was amplified from environmental DNA for all the stations using *rbc*L primers^24^. Subsequent steps including cloning, sequencing, pre-phylogenetic sequence analyses were undertaken based on published protocol^24^. A total of 148 *rbc*L sequences were generated from SBR and their GenBank accession numbers are KT335277-KT335427.

### Phylogenetic tree construction

Uncultured form ID *rbc*L amino acid sequences (184 amino acids length) were aligned with the representative of cultured chromophytic phytoplankton *rbc*L sequences in an online version of Clustal Omega (http://www.ebi.ac.uk/Tool/msa/clustalo). Sequences of insufficient length (< 125 amino acid length) were not considered in the final alignment. The form II *rbc*L sequence of *Lingulodinium polyedrum* (Acc. No. AAA98748) was chosen as outgroup. Poorly aligned positions and divergent regions of the alignment were removed in GBlocks^46^ using similarity matrices. The parameters used for GBlocks were minimum number of sequences for a conserved position; 964, minimum number of sequences for a flanking position; 964, maximum number of contiguous non-conserved positions; 8, and minimum length of a block; 5. The positions with a gap in less than 50% of the sequences were allowed in the final alignment. The new number of positions in final alignment was 178 (91% of the original 194 positions). Phylogenetic tree was constructed with RAxML v7.7.1 as implemented in vital IT unit of the Swiss Institute of Bioinformatics web server^47^ (http://embnet.vital-it.ch/raxml-bb/). GAMMA+P-Inver model of rate heterogeneity was estimated up to accuracy of 0.001 Log Likelihood units. The JTT model was used as substitution matrix based on the final alignment. The final ML optimization likelihood score was −35774.674391. The portion of gap and completely undetermined characters in the final alignment was only 3.77%. One hundred independent maximum likelihood (ML) inferences were run on the alignment and the best scoring ML tree was used as final tree. Different oceanographic ecoregions (based on sequences generated from different locations) were mapped onto the tree using interactive Tree of Life (iTOL) program^48^.

### Bioinformatics and Statistical analyses

The program MOTHUR^49^ v1.11.0 was used to determine the number of operational taxonomic units (OTUs) present in environmental form ID *rbc*L datasets at varying level of amino acid sequence identity. Rarefaction curves and beta-diversity matrices were generated from different ecoregions based on translated amino acid sequences. The AMOVA and ANOSIM analyses were conducted with 1000 permutations using distance matrices generated in MOTHUR. The LIBSHUFF analysis was also performed to test whether two or more environment types have the same structure of chromophytic phytoplankton in terms of OTU distribution using Cramer-von Mises test statistic in MOTHUR, using the default settings. Phylogenetic (P) significance test, UniFrac significance test, and Principal Coordinate Analysis (PCoA) were undertaken using FastUniFrac algorithm^50^ on the UniFrac website (http://bmf2.colorado.edu/fastunifrac/index.psp) using the best RAxML tree and an environmental file assigning each sequence to one of seven different ecoregions as input. Weighted normalized UniFrac distances were undertaken for P test, UniFrac significance test, and PCoA such that each dataset contributes equally to the distance calculated. Average pairwise identities of *rbc*L sequences were determined at amino acid level for each environmental type using Sequence Demarcation Tool^51^ (SDT) v1.2. Variation of G+C percentage in each dataset was calculated using BioEdit^52^ v7.0. Test for natural selection pressures on form ID *rbc*L sequences for each dataset were conducted using maximum likelihood-based SLAC methodology^53^ as implemented in the HyPhy package^54^ and run using web interface at http://www.datamonkey.org. For analyses of natural selection pressure within each ecoregion dataset, automatic nucleotide substitution model selection was undertaken before the SLAC analysis. The ratio of non-synonymous to synonymous substitutions (dN/dS) was calculated in each dataset separately at P < 0.1 significance level.

## Additional Information

**How to cite this article**: Samanta, B. and Bhadury, P. A comprehensive framework for functional diversity patterns of marine chromophytic phytoplankton using *rbc*L phylogeny. *Sci. Rep.* **6**, 20783; doi: 10.1038/srep20783 (2016).

## Supplementary Material

Supplementary Information

## Figures and Tables

**Table 1:** Summary of statistics of form ID *rbc*L sequence datasets at different amino acid identity level.

	Total sequences	Number of *rbc*L protein sequence OTUs (identity level)						
		Unique	99%	98%	97%	95%	90%	85%
Overall	2624	1112	923	654	493	319	105	38
East China Sea (EC)	712	294	252	183	142	100	38	17
Sundarbans (SB)	666	299	259	160	101	51	14	6
South China Sea (SC)	526	152	120	80	63	45	17	9
ALOHA (AL)	246	109	102	84	77	62	26	9
Gulf of Mexico (GM)	197	168	137	120	106	86	31	15
Monterey Bay (MB)	144	71	68	62	55	40	14	8
English Channel (L4)	133	64	63	53	49	36	15	5
