# Supplementary material for: A comprehensive framework for functional diversity patterns of marine chromophytic phytoplankton using rbcL phylogeny
Source: Sci Rep. 2016 Feb 10;6:20783. doi: 10.1038/srep20783 (PMC4748293; doi:10.1038/srep20783)
Supplement: Supplementary Information [file srep20783-s1.pdf]

Supplementary information

A comprehensive framework for functional diversity patterns of marine chromophytic phytoplankton using *rbc*L phylogeny

BRAJOGOPAL SAMANTA AND PUNYASLOKE BHADURY

Integrative Taxonomy and Microbial Ecology Research Group, Department of Biological Sciences, Indian Institute of Science Education and Research Kolkata, Mohanpur-741246, Nadia, West Bengal, India.

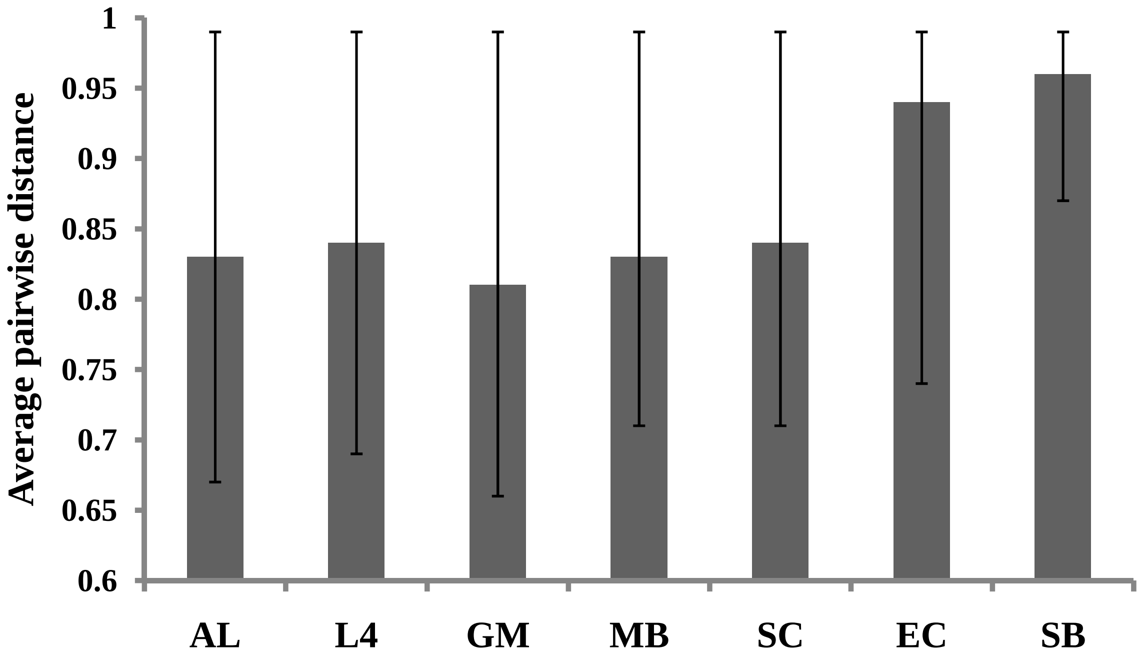

Figure S1: Average pairwise distance at the amino acid level. Error bars indicate the highest and lowest pairwise distance in each data set. AL = ALOHA, L4 = L4 site of Western English Channel, MB = Monterey Bay, GM = Gulf of Mexico, SC = South China Sea, EC = East China Sea, and SB = Sundarbans mangrove ecosystem.

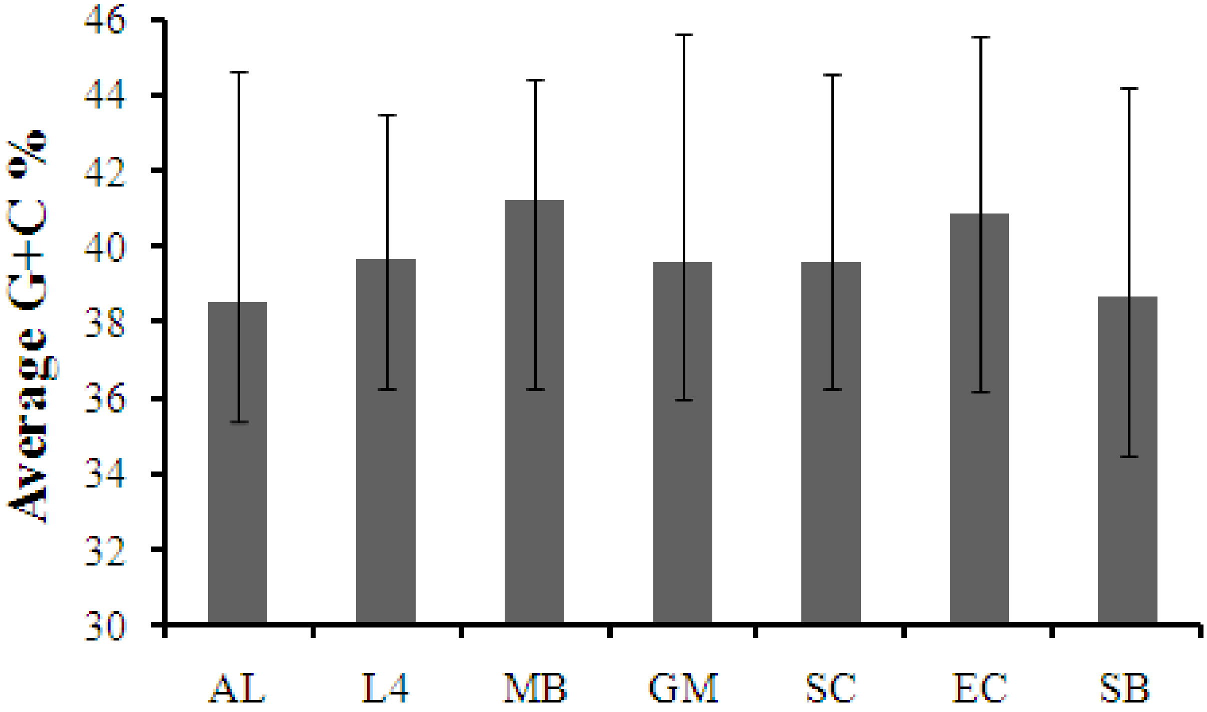

Figure S2: Average G+C percentage. Error bars indicate the highest and lowest G+C % in each data set. AL = ALOHA, L4 = L4 site of Western English Channel, MB = Monterey Bay, GM = Gulf of Mexico, SC = South China Sea, EC = East China Sea, and SB = Sundarbans mangrove ecosystem.

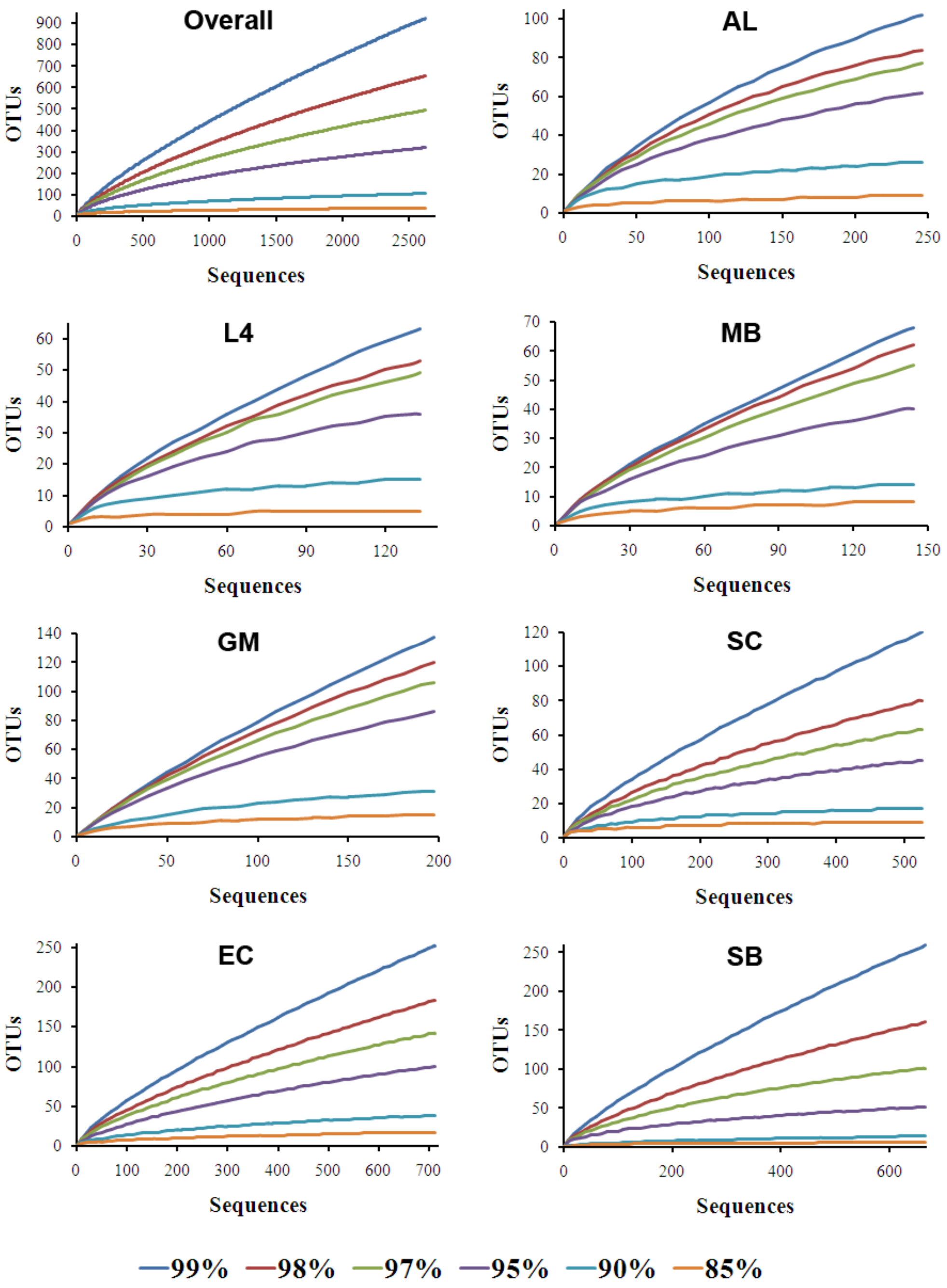

Figure S3: Rarefaction analyses of form ID *rbc*L sequences of each ecoregion datasets. Curves represent rarefaction analyses of OTUs defined by 99, 98, 97, 95, 90, and 85% identity at the amino acid level. AL = ALOHA, L4 = L4 site of Western English Channel, MB = Monterey Bay, GM = Gulf of Mexico, SC = South China Sea, EC = East China Sea, and SB = Sundarbans mangrove ecosystem.

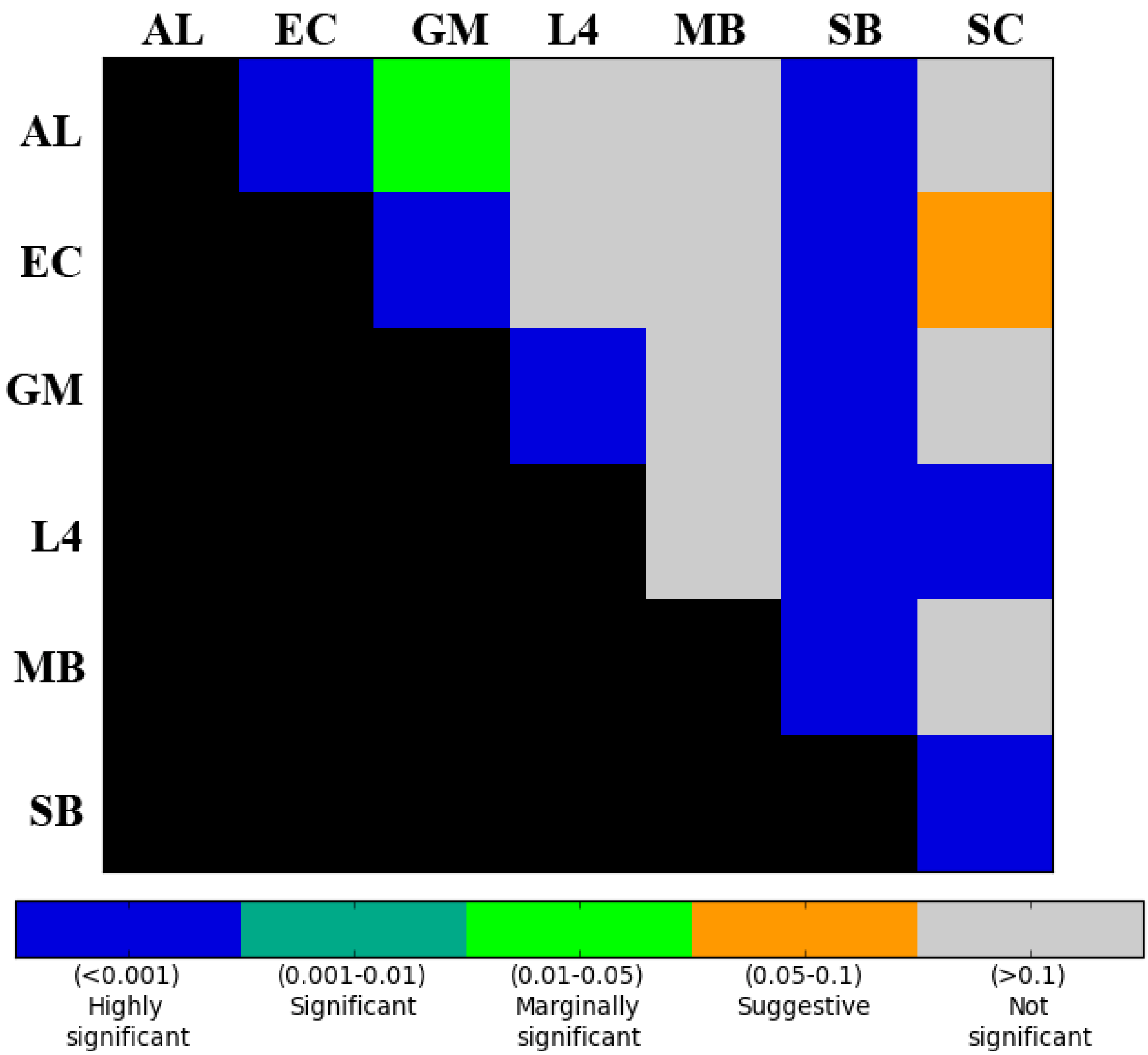

Figure S4: UniFrac significance test for each pair of environment type based on unique branch length present in each *rbc*L dataset. AL = ALOHA, L4 = L4 site of Western English Channel, MB = Monterey Bay, GM = Gulf of Mexico, SC = South China Sea, EC = East China Sea and SB = Sundarbans mangrove ecosystem.

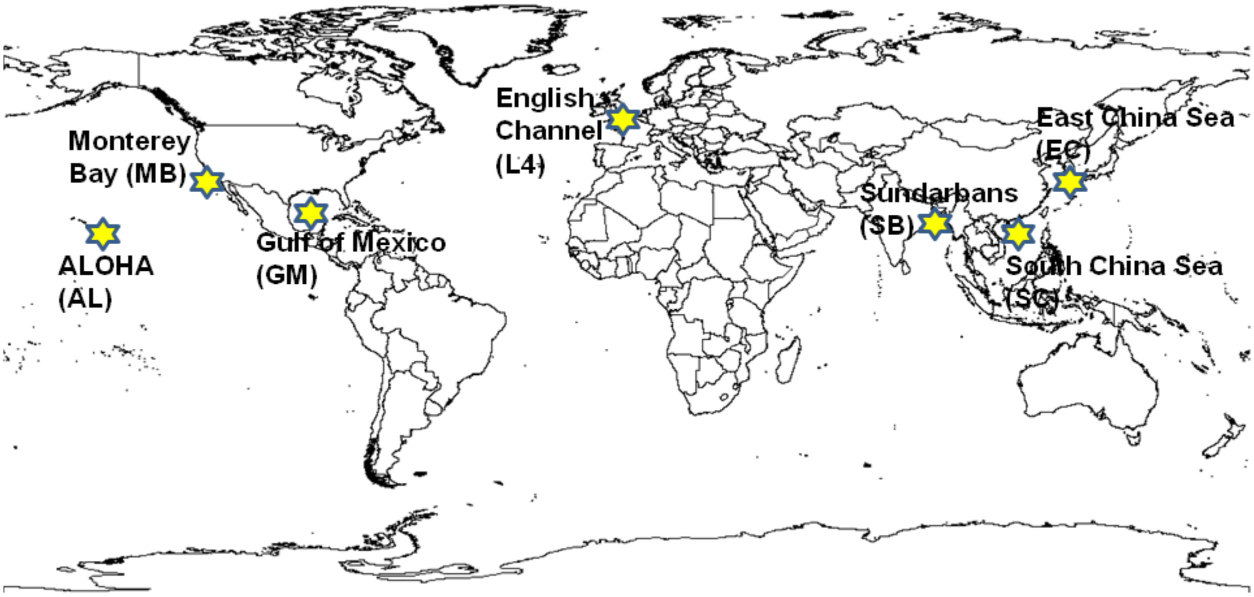

Figure S5: Schematic representation of the targeted locations of the world. Form ID *rbc*L sequences were retrieved from GenBank across seven different ecologically significant ecoregions of the world: ALOHA station (AL), English Channel (L4), Monterey Bay (MB), Gulf of Mexico (GM), South China Sea (SC), East China Sea (EC), and Sundarbans mangrove ecosystem (SB). Details of the each location and sampling strategies were given in the text method section. The map has been created using DIVA-GIS v7.5 (free computer program available at http://www.diva-gis.org/).

Table S1: Details of cultured chromophytic phytoplankton *rbc*L sequences were used in global phylogeny to annotate the uncultured formed ID *rbc*L sequences from different oceanographic ecoregions.

**Acc. No**	**Genus**	**Order**	**Class**
**ABF60369**	*Thalassiosira weissflogii*	Thalassiosirales	Bacillariophyceae
**AER38498**	*Thalassiosira conferta*	Thalassiosirales	Bacillariophyceae
**AGX25178**	*Roundia cardiophora*	Thalassiosirales	Bacillariophyceae
**AHE78130**	*Cyclotella meneghiniana*	Thalassiosirales	Bacillariophyceae
**ABF60389**	*Discostella stelligera*	Thalassiosirales	Bacillariophyceae
**AFO84266**	*Discostella* sp. RG-2012	Thalassiosirales	Bacillariophyceae
**ABF60391**	*Cyclotella pseudostelligera*	Thalassiosirales	Bacillariophyceae
**ABF60401**	*Stephanodiscus minutulus*	Thalassiosirales	Bacillariophyceae
**ABF60398**	*Cyclostephanos* sp. WTC18	Thalassiosirales	Bacillariophyceae
**ABF60372**	*Detonula pumila*	Thalassiosirales	Bacillariophyceae
**ABF60356**	*Bacterosira* sp. CCMP991	Thalassiosirales	Bacillariophyceae
**AEB91182**	*Planktoniella sol*	Thalassiosirales	Bacillariophyceae
**ABF60364**	*Thalassiosira mediterranea*	Thalassiosirales	Bacillariophyceae
**AGQ04195**	*Thalassiosira Sundarbana*	Thalassiosirales	Bacillariophyceae
**ABF60373**	*Thalassiosira punctigera*	Thalassiosirales	Bacillariophyceae
**AEB91183**	*Minidiscus trioculatus*	Thalassiosirales	Bacillariophyceae
**ABF60326**	*Porosira pseudodenticulata*	Thalassiosirales	Bacillariophyceae
**ABF6037**	*Shionodiscus ritscheri*	Thalassiosirales	Bacillariophyceae
**ABF60347**	*Thalassiosira eccentric*	Thalassiosirales	Bacillariophyceae
**AGR52758**	*Detonula confervacea*	Thalassiosirales	Bacillariophyceae
**ABN72968**	*Pseudo-nitzschia delicatissima*	Bacillariales	Bacillariophyceae
**ABN72951**	*Fragilariopsis cylindrus*	Bacillariales	Bacillariophyceae
**ABN72952**	*Fragilariopsis kerguelensis*	Bacillariales	Bacillariophyceae
**AEB91230**	*Denticula kuetzingii*	Bacillariales	Bacillariophyceae
**AGN91053**	*Ditylum brightwellii*	Lithodesmiales	Bacillariophyceae
**BAL43112**	*Nitzschia dubiiformis*	Bacillariales	Bacillariophyceae
**AGG86650**	*Nitzschia palea*	Bacillariales	Bacillariophyceae
**BAL43113**	*Psammodictyon constrictum*	Bacillariales	Bacillariophyceae
**AGN91086**	*Corethron hystrix*	Corethrales	Bacillariophyceae
**AGN91035**	*Achnanthes* sp. 1 MPA-2013	Achnanthales	Bacillariophyceae
**AEB91220**	*Tryblionella apiculata*	Bacillariales	Bacillariophyceae
**AGN91044**	*Bellerochea malleus*	Hemiaulales	Bacillariophyceae

**ABF60322**	*Helicotheca tamesis*	Lithodesmiales	Bacillariophyceae
**ABF60323**	*Lithodesmium undulatum*	Lithodesmiales	Bacillariophyceae
**ABF60379**	*Skeletonema menzellii*	Thalassiosirales	Bacillariophyceae
**AGN91101**	*Pseudosolenia calcar-avis*	Rhizosoleniales	Bacillariophyceae
**AGN91056**	*Hemiaulus sinensis*	Hemiaulales	Bacillariophyceae
**AII41580**	*Guinardia striata*	Rhizosoleniales	Bacillariophyceae
**AEB91218**	*Gyrosigma acuminatum*	Naviculales	Bacillariophyceae
**AEB91188**	*Minutocellus polymorphus*	Cymatosirales	Bacillariophyceae
**AGN91057**	*Extubocellulus* sp.	Cymatosirales	Bacillariophyceae
**AEB91191**	*Extubocellulus cribriger*	Cymatosirales	Bacillariophyceae
**AEB91250**	*Papiliocellulus simplex*	Cymatosirales	Bacillariophyceae
**AGN91059**	*Minutocellus polymorphus*	Cymatosirales	Bacillariophyceae
**AEB91189**	*Arcocellulus mammifer*	Cymatosirales	Bacillariophyceae
**AGR52875**	*Thalassionema* sp. 2BOF	Thalassionemales	Bacillariophyceae
**AGZ17067**	*Thalassionema frauenfeldii*	Thalassionemales	Bacillariophyceae
**CCF79523**	*Haslea ostrearia*	Naviculales	Bacillariophyceae
**AGN91099**	*Proboscia* sp. 1 MPA-2013	Rhizosoleniales	Bacillariophyceae
**AEB91217**	*Diploneis subovalis*	Naviculales	Bacillariophyceae
**AAL13214**	*Bolidomonas mediterranea*		Bolidophyceae
**AAL26316**	*Bolidomonas pacifica*		Bolidophyceae
**AEB91197**	*Urosolenia eriensis*	Rhizosoleniales	Bacillariophyceae
**AII41566**	*Asteromphalus* sp. TN-2014	Asterolamprales	Bacillariophyceae
**AGN91096**	*Guinardia flaccid*	Rhizosoleniales	Bacillariophyceae
**AEB91234**	*Stellarima microtrias*	Coscinodiscales	Bacillariophyceae
**AEB91255**	*Centronella reicheltii*	Fragilariales	Bacillariophyceae
**AFD04477**	*Rhabdonema arcuatum*	Rhabdonematales	Bacillariophyceae
**AEB91231**	*Ctenophora pulchella*	Fragilariales	Bacillariophyceae
**BAL43103**	*Pteroncola inane*	Fragilariales	Bacillariophyceae
**AGC59938**	*Amphitetras antediluviana*	Triceratiales	Bacillariophyceae
**AGN91080**	*Triceratium dubium*	Triceratiales	Bacillariophyceae
**AGN91067**	*Pseudauliscus peruvianus*	Triceratiales	Bacillariophyceae
**AGN91048**	*Cerataulus smithii*	Triceratiales	Bacillariophyceae
**AGN91078**	*Odontella* sp. 1 MPA-2013	Eupodiscales	Bacillariophyceae
**AEB91295**	*Mastodiscus radiatus*	Triceratiales	Bacillariophyceae
**AGN91066**	*Pleurosira laevis f. polymorpha*	Triceratiales	Bacillariophyceae
**AEB91285**	*Amphitetras antediluviana*	Triceratiales	Bacillariophyceae
**AEB91291**	*Palmerina hardmaniana*	Coscinodiscales	Bacillariophyceae
**AGN91089**	*Coscinodiscus radiatus*	Coscinodiscales	Bacillariophyceae
**BAL43101**	*Pseudohimantidium pacificum*	Protoraphidales	Bacillariophyceae
**AGZ17070**	*Hyalosynedra* sp. 1 CSL-2013	Fragilariales	Bacillariophyceae

**AEB91303**	*Hydrosera* sp. CYTX025	Biddulphiales	Bacillariophyceae
**AFD04471**	*Florella pascuensis*	Striatellales	Bacillariophyceae
**AFD04468**	*Cyclophora tabellariformis*	Cyclophorales	Bacillariophyceae
**AII41576**	*Cyclophora* sp. TN-2014	Cyclophorales	Bacillariophyceae
**AGN91068**	*Stictocyclus stictodiscus*	Stictocyclales	Bacillariophyceae
**AEB91304**	*Isthmia enervis*	Biddulphiales	Bacillariophyceae
**ADQ12897**	*Perideraion montgomeryi*	Fragilariales	Bacillariophyceae
**ADQ12896**	*Koernerella recticostata*	Fragilariales	Bacillariophyceae
**AEB91242**	*Diatoma tenue*	Fragilariales	Bacillariophyceae
**BAL43090**	*Diatoma moniliforme*	Fragilariales	Bacillariophyceae
**AII41596**	*Tetracyclus* sp. TN-2014	Tabellariales	Bacillariophyceae
**BAL43106**	*Tabularia laevis*	Fragilariales	Bacillariophyceae
**AGZ17071**	*Licmosphenia peragallioides*		Bacillariophyceae
**AII41583**	*Licmophora normaniana*		Bacillariophyceae
**AHE78123**	*Fistulifera saprophila*	Naviculales	Bacillariophyceae
**BAL43109**	*Campylodiscus thuretii*	Surirellales	Bacillariophyceae
**AEB39383**	*Stenopterobia curvula*	Surirellales	Bacillariophyceae
**AEB91278**	*Surirella minuta*	Surirellales	Bacillariophyceae
**AEB91279**	*Cymatopleura elliptica*	Surirellales	Bacillariophyceae
**AEB91201**	*Craticula cuspidata*	Naviculales	Bacillariophyceae
**AET10441**	*Phaeodactylum tricornutum*	Naviculales	Bacillariophyceae
**AEB91224**	*Pinnularia brebissonii*	Naviculales	Bacillariophyceae
**AEB91211**	*Neidium bisulcatum*	Naviculales	Bacillariophyceae
**AFX61740**	*Parlibellus delognei f. ellipticus*	Naviculales	Bacillariophyceae
**AEB91229**	*Scoliopleura peisonis*	Naviculales	Bacillariophyceae
**AFX61710**	*Berkeleya* sp. 9SEH	Naviculales	Bacillariophyceae
**AGN91036**	*Amphipleura pellucida*	Naviculales	Bacillariophyceae
**AEB91225**	*Fallacia pygmaea*	Naviculales	Bacillariophyceae
**AEB91309**	*Aulacodiscus* sp.	Coscinodiscales	Bacillariophyceae
**AGN91037**	*Climaconeis undulata*	Naviculales	Bacillariophyceae
**AEB91199**	*Stauroneis acuta*	Naviculales	Bacillariophyceae
**CCF79527**	*Haslea crucigera*	Naviculales	Bacillariophyceae
**ACJ74403**	*Entomoneis cf. alata*	Naviculales	Bacillariophyceae
**AEB39378**	*Entomoneis ornata*	Naviculales	Bacillariophyceae
**CAM97887**	*Eolimna minima*	Naviculales	Bacillariophyceae
**AEB91216**	*Fallacia monoculata*	Naviculales	Bacillariophyceae
**AEB91212**	*Cocconeis placentula*	Achnanthales	Bacillariophyceae
**AAT78580**	*Petroneis humerosa*	Lyrellales	Bacillariophyceae
**AAT78579**	*Lyrella* sp. E3461	Lyrellales	Bacillariophyceae
**AEB91246**	*Lemnicola hungarica*	Achnanthales	Bacillariophyceae

**ABO77825**	*Rossia* sp. E3333	Naviculales	Bacillariophyceae
**AEB91227**	*Placoneis elginensis*	Cymbellales	Bacillariophyceae
**CAM97959**	*Encyonema caespitosum*	Cymbellales	Bacillariophyceae
**AFX81366**	*Planothidium lanceolatum*	Achnanthales	Bacillariophyceae
**AFV95051**	*Gomphonema parvulum*		Bacillariophyceae
**AHK61270**	*Diprora haenaensis*	Cymbellales	Bacillariophyceae
**AGT21406**	*Sellaphora pupula*	Naviculales	Bacillariophyceae
**AEB91281**	*Podocystis spathulata*	Fragilariales	Bacillariophyceae
**AET10428**	*Aulacoseira granulata*		Bacillariophyceae
**AER38496**	*Asterionella glacialis*	Fragilariales	Bacillariophyceae
**AGN91103**	*Rhizosolenia setigera*	Rhizosoleniales	Bacillariophyceae
**AGG86639**	*Mayamaea permitis*	Naviculales	Bacillariophyceae
**YP_009029287**	*Leptocylindrus danicus*	Leptocylindrales	Bacillariophyceae
**AHX02827**	*Halamphora oligotraphenta*	Naviculales	Bacillariophyceae
**AHX02804**	*Amphora caribaea*	Thalassiophysales	Bacillariophyceae
**AGG86629**	*Amphora montana*	Thalassiophysales	Bacillariophyceae
**AGN91040**	*Mastogloia* sp.	Mastogloiales	Bacillariophyceae
**AEB39374**	*Rhopalodia gibba*	Rhopalodiales	Bacillariophyceae
**AEB39377**	*Epithemia turgida*	Rhopalodiales	Bacillariophyceae
**AII41589**	*Parlibellus hamulifer*	Naviculales	Bacillariophyceae
**AGN91041**	*Meuniera membranacea*	Naviculales	Bacillariophyceae
**BAL43111**	*Navicula* sp. s0020	Naviculales	Bacillariophyceae
**AEB91223**	*Navicula* sp.	Naviculales	Bacillariophyceae
**AEB39371**	*Hantzschia amphioxys var. major*	Bacillariales	Bacillariophyceae
**AEB91247**	*Bacillaria paxillifer*	Bacillariales	Bacillariophyceae
**AGN91050**	*Cymatosira belgica*	Cymatosirales	Bacillariophyceae
**AGN91045**	*Biddulphia cf. reticulum*	Biddulphiales	Bacillariophyceae
**AGN91049**	*Cymatosira lorenziana*	Cymatosirales	Bacillariophyceae
**AER38496**	*Asterionella glacialis*	Fragilariales	Bacillariophyceae
**AEB91236**	*Synedropsis cf. recta*	Fragilariales	Bacillariophyceae
**BAL43106**	*Tabularia laevis*	Fragilariales	Bacillariophyceae
**AGG86653**	*Ulnaria ulna*	Fragilariales	Bacillariophyceae
**AEB91235**	*Tabularia cf. tabulata*	Fragilariales	Bacillariophyceae
**BAL43107**	*Thalassiothrix longissima*	Thalassionemales	Bacillariophyceae
**AEB91277**	*Stephanopyxis turris*	Melosirales	Bacillariophyceae
**AGN91051**	*Dactyliosolen blavyanus*	Rhizosoleniales	Bacillariophyceae
**BAL43105**	*Striatella unipunctata*	Striatellales	Bacillariophyceae
**AGN03042**	*Striatella unipunctata*	Striatellales	Bacillariophyceae
**AEB91240**	*Staurosirella pinnata*	Fragilariales	Bacillariophyceae
**AEB91198**	*Nanofrustulum cf. shiloi*	Fragilariales	Bacillariophyceae

**AGX25177**	*Brockmanniella brockmannii*	Cymatosirales	Bacillariophyceae
**AGN91065**	*Plagiogrammopsis vanheurckii*	Cymatosirales	Bacillariophyceae
**AGN91046**	*Cerataulina pelagica*	Hemiaulales	Bacillariophyceae
**AGN91055**	*Eucampia zodiacus*	Hemiaulales	Bacillariophyceae
**AER38507**	*Rhizochromulina marina*	Rhizochromulinales	Dictyochophyceae
**BAC16244**	*Ciliophrys infusionum*	Pedinellales	Dictyochophyceae
**AER38504**	*Pseudopedinella elastica*	Pedinellales	Dictyochophyceae
**BAC16243**	*Pteridomonas danica*	Pedinellales	Dictyochophyceae
**AER38503**	*Apedinella radians*	Pedinellales	Dictyochophyceae
**CAO98894**	*Pseudochattonella verruculosa*	Florenciellales	Dictyochophyceae
**AAK85735**	*Dictyocha speculum*	Dictyochales	Dictyochophyceae
**AGN91098**	*Melosira varians*	Melosirales	Bacillariophyceae
**AGN91082**	*Actinocyclus* sp. 1 MPA-2013	Coscinodiscales	Bacillariophyceae
**AGN91085**	*Actinoptychus undulatus*	Coscinodiscales	Bacillariophyceae
**AGR52702**	*Actinoptychus senarius*	Coscinodiscales	Bacillariophyceae
**AEB91273**	*Ardissonea formosa*	Fragilariales	Bacillariophyceae
**ADE09920**	*Toxarium undulatum*	Toxariales	Bacillariophyceae
**AGN91043**	*Synedra fulgens*	Fragilariales	Bacillariophyceae
**AGN91094**	*Endictya oceanica*	Melosirales	Bacillariophyceae
**AEB91269**	*Hyalodiscus* sp. ECT3681	Melosirales	Bacillariophyceae
**AGN91097**	*Hyalodiscus* sp. 1 MPA-2013	Melosirales	Bacillariophyceae
**AEB91178**	*Chaetoceros muellerii*	Chaetocerotales	Bacillariophyceae
**BAL43091**	*Dimeregramma minor var. nanum*	Triceratiales	Bacillariophyceae
**AER38534**	*Heterosigma akashiwo*	Chattonellales	Raphidophyceae
**YP_001936308**	*Heterosigma akashiwo*	Chattonellales	Raphidophyceae
**BAJ11797**	*Chattonella marina var. antiqua*	Chattonellales	Raphidophyceae
**AER38532**	*Chattonella subsalsa*	Chattonellales	Raphidophyceae
**AER38531**	*Chattonella marina*	Chattonellales	Raphidophyceae
**BAF80664**	*Fibrocapsa japonica*	Chattonellales	Raphidophyceae
**BAB20786**	*Imantonia rotunda*	Prymnesiales	Haptophyceae
**BAA96248**	*Haptolina hirta*	Prymnesiales	Haptophyceae
**YP_008145509**	*Phaeocystis globosa*	Phaeocystales	Haptophyceae
**YP_005088729**	*Phaeocystis antarctica*	Phaeocystales	Haptophyceae
**BAA96246**	*Gephyrocapsa oceanica*	Isochrysidales	Haptophyceae
**BAA96247**	*Emiliania huxleyi*	Isochrysidales	Haptophyceae
**BAB20783**	*Isochrysis galbana*	Isochrysidales	Haptophyceae
**BAB20782**	*Helicosphaera carteri*	Zygodiscales	Haptophyceae

**BAB20790**	*Chrysocampanula spinifera*	Prymnesiales	Haptophyceae
**BAA96245**	*Umbilicosphaera sibogae var. foliosa*	Coccolithales	Haptophyceae
**BAA96244**	*Calyptrosphaera sphaeroidea*	Coccosphaerales	Haptophyceae
**AET10438**	*Coccolithus pelagicus*	Coccolithales	Haptophyceae
**BAB20780**	*Calcidiscus leptoporus*	Coccolithales	Haptophyceae
**BAB20784**	*Chrysochromulina parva*	Prymnesiales	Haptophyceae
**BAB20788**	*Prymnesium parvum*	Prymnesiales	Haptophyceae
**BAB20789**	*Platychrysis* sp. TKB8934	Prymnesiales	Haptophyceae
**BAB20779**	*Cruciplacolithus neohelis*	Coccolithales	Haptophyceae
**AGE93739**	*Pavlova lutheri*	Pavlovales	Haptophyceae
**BAA96249**	*Rebecca salina*	Pavlovales	Haptophyceae
**BAD42424**	Teleaulax sp. TUC-2	Pyrenomonadales	Cryptophyceae
**BAD42425**	*Plagioselmis* sp. TUC-1	Cryptomonadales	Cryptophyceae
**BAD42427**	*Geminigera cryophila*	Pyrenomonadales	Cryptophyceae
**BAD42426**	*Proteomonas sulcata*	Pyrenomonadales	Cryptophyceae
**YP_001293518**	*Rhodomonas_salina*	Pyrenomonadales	Cryptophyceae
**AAM62090**	*Pyrenomonas helgolandii*	Pyrenomonadales	Cryptophyceae
**AAQ04824**	*Storeatula major*	Pyrenomonadales	Cryptophyceae
**AAM62089**	*Chroomonas* sp. SAG 980-1	Pyrenomonadales	Cryptophyceae
**NP_050705**	*Guillardia theta*	Cryptomonadales	Cryptophyceae
**YP_003359220**	*Cryptomonas paramecium*	Cryptomonadales	Cryptophyceae
**ABV21891**	*Chroodactylon ornatum*	Porphyridiales	Rhodo(Bangiophyceae)
**BAO23622**	*Porphyridium purpureum*	Porphyridiales	Rhodo(Bangiophyceae)
**ABC33844**	*Erythrolobus coxiae*	Porphyridiales	Rhodo(Bangiophyceae)
**YP_008519840**	*Nannochloropsis salina*	Eustigmatales	Eustigmatophyceae
**AER38513**	*Nannochloropsis oculata*	Eustigmatales	Eustigmatophyceae
**AAC02914**	*Eustigmatos magnus*	Eustigmatales	Eustigmatophyceae
**AER38517**	*Vischeria stellata*	Eustigmatales	Eustigmatophyceae
**AAF00606**	*Chromulina nebulosa*	Chromulinales	Chrysophyceae
**ABN46945**	*Ochromonas vasocystis*	Chromulinales	Chrysophyceae
**ABN46918**	*Dinobryon cylindricum*	Chromulinales	Chrysophyceae
**AAC02909**	*Chrysolepidomonas dendrolepidota*	Chromulinales	Chrysophyceae
**ABN46910**	*Chrysocapsa paludosa*	Chromulinales	Chrysophyceae
**AAF00607**	*Chrysocapsa vernalis*	Chromulinales	Chrysophyceae
**AER38502**	*Ochromonas tuberculata*	Chromulinales	Chrysophyceae
**AIG21868**	*Chrysopodocystis socialis*	Hibberdiales	Chrysophyceae
**ABN46911**	*Chrysonebula flava*	Hydrurales	Chrysophyceae
**AAM45880**	*Phaeomonas parva*	Pinguiochrysidales	Pinguiophyceae
**AAM45878**	*Pinguiococcus pyrenoidosus*	Pinguiochrysidales	Pinguiophyceae

**AAM45877**	*Glossomastix chrysoplasta*	Pinguiochrysidales	Pinguiophyceae
**AAM45879**	*Polypodochrysis teissieri*	Pinguiochrysidales	Pinguiophyceae
**AER38518**	*Ankylochrysis lutea*	Sarcinochrysidales	Pelagophyceae
**AER38519**	*Aureococcus anophagefferens*	Pelagomonadales	Pelagophyceae
**AER38522**	*Pelagococcus subviridis*	Pelagomonadales	Pelagophyceae
**AAC02816**	*Pelagomonas calceolata*	Pelagomonadales	Pelagophyceae
**AAD39124**	*Aureoumbra lagunensis* CCMP1510	Pelagomonadales	Pelagophyceae
**AAC02923**	*Sarcinochrysis marina* CCMP770	Pelagomonadales	Pelagophyceae
**AAC02922**	*Pulvinaria* sp. CCMP292	Sarcinochrysidales	Pelagophyceae
**AAC62465**	*Pleurochloridella botrydiopsis*	Pleurochloridellales	Phaeothamniophyceae
**AAF00605**	*Tetrasporopsis fuscescens*	Phaeothamniales	Phaeothamniophyceae
**AAC62462**	*Phaeothamnion confervicola*	Phaeothamniales	Phaeothamniophyceae
**AAC62463**	*Phaeoschizochlamys mucosa*	Phaeothamniales	Phaeothamniophyceae
**AAF00604**	*Stichogloea globosa*	Phaeothamniales	Phaeothamniophyceae
**AAC62459**	*Botrydium stoloniferum*	Botrydiales	Xanthophyceae
**AAC62460**	*Mischococcus sphaerocephalus*	Mischococcales	Xanthophyceae
**ABN46960**	*Tessellaria volvocina*	Synurales	Synurophyceae
**ABN46959**	*Mallomonas insignis*	Synurales	Synurophyceae
**AER38535**	*Mallomonas caudata*	Synurales	Synurophyceae
**ABN46957**	*Synura curtispina*	Synurales	Synurophyceae
**AER38536**	*Synura petersenii*	Synurales	Synurophyceae
**BAJ72270**	*Triparma* sp. TOY-0807	Parmales	Synurophyceae

Table S2: Taxonomic classes of chromophytic phytoplankton were detected for seven targeted ecoregions based on global *rbc*L phylogeny. AL = ALOHA, L4 = L4 site of Western English Channel, MB = Monterey Bay, GM = Gulf of Mexico, SC = South China Sea, EC = East China Sea and SB = Sundarbans mangrove ecosystem.

Classes	Bacillariophyceae	Cryptophyceae	Haptophyceae	Pelagophyceae	Dictyochophyceae	Chrysophyceae
**AL**	√	√	√	√		
**L4**	√	√	√		√	
**MB**	√	√	√	√	√	√
**GM**	√	√	√	√	√	√
**SC**	√	√	√		√	√
**EC**	√	√	√	√	√	√
**SB**	√	√	√	√		
Classes	Eusigmatophyceae	Raphidophyceae	Rhodophyceae	Bolidophyceae	Pinguiophyceae	
**AL**						
**L4**				√		
**MB**		√		√		
**GM**	√		√		√	
**SC**	√	√				
**EC**	√	√	√			
**SB**	√	√				

Table S3: The ANOSIM and AMOVA results of the total *rbc*L dataset for pairwise comparisons. AL = ALOHA, L4 = L4 site of Western English Channel, MB = Monterey Bay, GM = Gulf of Mexico, SC = South China Sea, EC = East China Sea, and SB = Sundarbans mangrove ecosystem.

	ANOSIM		AMOVA	
**Comparison**	**R-value**	**P-value**	**Fs-value**	**P-value**
**AL-EC-GM-L4-MB-SB-SC**	0.359335	<0.001	1.06672e+006	<0.001
**AL-EC**	1.00006	<0.001	2.38752e+006	<0.001
**AL-GM**	1	<0.001	2.16546e+006	<0.001
**AL-L4**	1	<0.001	1.55593e+006	<0.001
**AL-MB**	1	<0.001	2.27772e+006	<0.001
**AL-SB**	1	<0.001	7.53347e+006	<0.001
**AL-SC**	1	<0.001	6.59428e+006	<0.001
**EC-GM**	0.204827	<0.001	44.9524	<0.001
**EC-L4**	0.121925	<0.001	27.432	<0.001
**EC-MB**	0.155383	<0.001	45.7813	<0.001
**EC-SB**	0.166456	<0.001	179.867	<0.001
**EC-SC**	0.122583	<0.001	127.838	<0.001
**GM-L4**	0.03845	0.013	15.0019	<0.001
**GM-MB**	0.015451	0.036	8.50455	<0.001
**GM-SB**	0.472156	<0.001	152.313	<0.001
**GM-SC**	0.236833	<0.001	34.0873	<0.001
**L4-MB**	0.042	<0.001	7.1927	<0.001
**L4-SB**	0.462543	<0.001	43.3008	<0.001
**L4-SC**	0.184512	<0.001	24.076	<0.001
**MB-SB**	0.53672	<0.001	129.496	<0.001
**MB-SC**	0.146999	<0.001	23.5278	<0.001
**SB-SC**	0.261507	<0.001	225.401	<0.001

Table S4: β-diversity analyses among seven targeted ecoregions at different similarity level of amino acid. AL = ALOHA, L4 = L4 site of Western English Channel, MB = Monterey Bay, GM = Gulf of Mexico, SC = South China Sea, EC = East China Sea, and SB = Sundarbans mangrove ecosystem.

1% cutoff							
	AL	EC	GM	L4	MB	SB	SC
AL		0	0	0	0	0	0
EC	0		0.809	0.951	0.83	0.843	0.872
GM	0	0.718		0.981	0.945	0.964	0.841
L4	0	0.822	0.959		0.87	0.996	0
MB	0	0.548	0.907	0.825		0.986	0.996
SB	0	0.757	0.923	0.977	0.916		0.962
SC	0	0.728	0.805	0.996	0.97	0.911	
2% cutoff							
3% cutoff							
	AL	EC	GM	L4	MB	SB	SC
AL		0	0	0	0	0	0
EC	0		0.467	0.691	0.347	0.555	0.691
GM	0	0.57		0.918	0.86	0.844	0.669
L4	0	0.503	0.753		0.786	0.965	0.978
MB	0	0.42	0.678	0.712		0.9	0.888
SB	0	0.487	0.65	0.738	0.707		0.835
SC	0	0.712	0.478	0.839	0.767	0.565	
5% cutoff							
10% cutoff							
	AL	EC	GM	L4	MB	SB	SC
AL		0	0	0	0	0	0
EC	0		0.08	0.142	0.08	0.352	0.413
GM	0	0.056		0.428	0.379	0.46	0.249
L4	0	0.057	0.237		0.405	0.446	0.651
MB	0	0.054	0.206	0.091		0.153	0.557
SB	0	0.048	0.194	0.12	0.139		0.35
SC	0	0.385	0.132	0.462	0.46	0.043	
15% cutoff							

Table S5: Coordinates of the sampling locations and the numbers of clones sequenced per library of Sundarbans Biosphere Reserve (SBR) were given in details.

**Clone library**	**Sampling Coordinates**	**Number of clones sequenced**
Stn18	22°07'15.1"N 88°57'06.00"E	19
Stn25	21°56'50.9"N 88°52'37.9"E	09
Stn29	21°49'45.37"N 88°53'00.42"E	13
Stn31	22°07'29.8"N 88°59'42.40"E	12
Stn35	21°47'35.27"N 88°55'19.11"E	12
Stn40 (2)	22°05'06.2"N 88°51'57.8"E	04
Stn40(3)	22°01'32.2"N 88°58'19.0"E	12
Stn55	21°54'52.52"N 88°53'11.37"E	13
Stn57	22°05'51.7"N 88°57'19.3"E	08
Stn64	21°43'40.24"N 88°54'37.84"E	12
Stn65	21°59'19.3"N 88°58'12.10"E	09
Stn71	21°40'28.87"N 88°57'28.58"E	25
